# Uric Acid Promotes Human Umbilical Vein Endothelial Cell Senescence In Vitro

**DOI:** 10.3390/metabo15060402

**Published:** 2025-06-14

**Authors:** Katarzyna Lewandowska, Justyna Mikuła-Pietrasik, Krzysztof Książek, Andrzej Tykarski, Paweł Uruski

**Affiliations:** 1 Department of Hypertensiology, Angiology and Internal Medicine, Poznan University of Medical Sciences, Długa 1/2 Str., 61-848 Poznan, Poland; tykarski@ump.edu.pl (A.T.); puruski@ump.edu.pl (P.U.); 2 Doctoral School, Poznan University of Medical Sciences, 60-812, Poznan, Poland; 3 Department of Pathophysiology of Ageing and Civilization Diseases, Poznan University of Medical Sciences, Święcickiego 4 Str., 60-781 Poznan, Poland; jmikula@ump.edu.pl; 4 Independent Researcher, 60-131 Poznań, Poland; kksiazek76@gmail.com

**Keywords:** uric acid, cellular senescence, HUVECs

## Abstract

Background/Objectives: Uric acid can act as a prooxidant or an antioxidant; therefore, its effects on human umbilical vein endothelial cells (HUVECs) were investigated to better understand its role in promoting cellular senescence and vascular dysfunction. Methods: HUVECs were exposed to different concentrations of exogenous uric acid levels typically found in patients with cardiovascular conditions (5 mg/dL, 7.5 mg/dL, and 10 mg/dL) to assess cell viability, proliferation, and senescence markers including SA-β-Gal activity, γ-H2A.X and 53BP1 expression, as well as mitochondrial dysfunction parameters such as reactive oxygen species (ROS) production, mitochondrial mass, and mitochondrial membrane potential (ΔΨm). Additionally, the secretion of factors related to the senescence-associated secretory phenotype (SASP) was quantified. Results: Uric acid concentrations of 7.5 mg/dL and above significantly reduced HUVEC viability, enhanced proliferation, and increased markers of cellular senescence, including SA-β-Gal activity and γ-H2A.X/53BP1 expression. Higher uric acid levels also led to increased ROS production, increased mitochondrial mass, and reduced membrane potential. Uric acid also dose-dependently increased IL-6, IL-8, HGF, GRO-1, and TGF-β1 levels. Conclusions: High uric acid concentrations (≥7.5 mg/dL) promote HUVEC senescence, possibly due to ROS-induced DNA damage. In addition, uric acid triggers the production of pro-inflammatory cytokines and growth factors.

## 1. Introduction

Uric acid (UA) is the final product of purine transformation. It acts as an antioxidant under normal concentrations, protecting cells from oxidative stress. The oxidation of hypoxanthine to xanthine and then to UA is catalyzed by xanthine dehydrogenase, which in hypoxic conditions converts to xanthine oxidase (XO), producing reactive oxygen species (ROS). Therefore, in hypoxic conditions, UA synthesis can also generate ROS, increasing oxidative stress. Moreover, the protective effect of UA cannot neutralize all ROS generated during XO-mediated synthesis [1,2,3]. The opposing biological effects of UA resulting from its concentration-dependent switch from anti to prooxidant properties are described as the UA paradox, with low UA concentrations exerting antioxidative activity, whereas high UA concentrations increase oxidative stress [4].

Elevated UA levels in the normal range (more than 5–6 mg/dL) or hyperuricemia (exceeding 7.0 mg/dL in men and 6.0 mg/dL in women) are linked to atherosclerosis and various cardiovascular diseases (CVDs) like arterial hypertension or ischemic heart disease [5]. The European Society of Hypertension (ESH) has identified UA as an independent risk factor for CVDs and advises regular monitoring of serum UA levels. Experts recommend a target serum UA level of less than 5.0 mg/dL in this population. Higher values are considered harmful [6,7].

**Figure 1:**
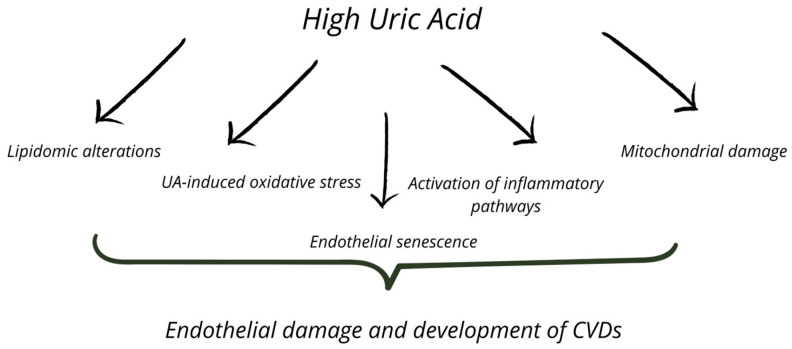
The selected interconnections between the various mechanisms connecting high UA levels and endothelial dysfunction.

High UA levels may induce endothelial dysfunction through increased oxidative stress [7]. Hypoxia can initially cause cell damage and trigger inflammation in CVDs [8]. In response, cells produce UA as a free radical scavenger [9], but inflammation and hypoxia may promote XO activation. Cell damage can occur through UA, which activates inflammatory pathways at high concentrations, XO, and mitochondrial respiratory chain damage, all of which promote ROS production [3,10,11,12,13,14,15]. An additional mechanism promoting endothelial cell damage may involve the detrimental impact of elevated UA levels on lipidome alterations [16]. The presence of multiple mechanisms contributing to cell damage poses a significant challenge for researchers and highlights that there is still much to explore in the context of UA and CVDs. Figure 1 illustrates the selected interconnections between the various mechanisms connecting high UA levels and endothelial dysfunction.

A possible common factor linking the negative effects of UA and the development of CVDs is endothelial damage via cellular senescence. UA may indirectly affect endothelial dysfunction via XO or directly impact vascular endothelial cell senescence.

Cellular senescence can arise through various mechanisms and proceed via distinct molecular pathways. Multiple forms of senescence exist, and they can be characterized using different molecular and cellular markers. One of the classical, although still widely used markers, is senescence-associated β-galactosidase (SA-β-Gal) activity, which increases in senescent cells due to enhanced lysosomal content. Additional markers include 53BP1 and γH2AX, which are associated with the DNA damage response (DDR) and indicate the presence of DNA double-strand breaks, a common feature in senescent cells [5].

A key feature of senescent cells is the development of a senescence-associated secretory phenotype (SASP), characterized by increased secretion of pro-inflammatory cytokines and chemokines, including IL-6, IL-8, GRO-1, and TGF-β1, among others [17].

The literature also describes so-called anti-inflammatory interleukins, one of which is IL-37. It inhibits the production of other pro-inflammatory cytokines, thereby modulating inflammation triggered by, e.g., elevated uric acid (UA) levels [18].

Mitochondrial dysfunction can be assessed by increased mitochondrial reactive oxygen species (ROS) production, detectable with MitoSOX fluorescence, decreased mitochondrial membrane potential, measured by JC-1 dye, and increased mitochondrial mass, evaluated using Nonyl Acridine Orange (NAO). An elevated signal from dihydrorhodamine 123 (DHR123) indicates increased cytosolic oxidative stress, reflecting a broader redox imbalance often seen in senescent cells [19].

Some studies have attempted to assess the role of UA in cellular senescence. There is growing evidence that cellular senescence of human umbilical vein endothelial cells (HUVECs) is an adverse effect of elevated uric acid (UA) levels. However, existing studies in this field present several limitations. In many cases, the assessment of senescence was based on an insufficient number of senescence markers. Additionally, the duration of UA exposure was often limited to a short incubation period of a maximum of 48 h, which may not adequately reflect the chronic nature of hyperuricemia. Some studies utilized supraphysiological concentrations of UA, which do not accurately represent levels observed in human serum [20,21,22].

In light of these limitations, we aimed to comprehensively evaluate the impact of UA on cellular senescence in HUVECs in vitro by (1) employing a broader panel of senescence markers, (2) extending the duration of exposure to reflect more physiologically relevant timeframes, and (3) using UA concentrations corresponding to those observed in patients. Furthermore, we sought to investigate potential mechanisms underlying UA-induced endothelial cell senescence.

## 2. Materials and Methods

### 2.1. Materials

Unless otherwise stated, all reagents were purchased from Sigma-Aldrich Corp. (St. Louis, MO, USA).

### 2.2. Cell Culture and Exposure to Exogenous UA

HUVECs were purchased from Lonza (Walkersville, MD, USA). Commercially purchased ampules of Human Umbilical Vein Endothelial Cells were derived from pooled donors. The cells were cultured in EBM™-2 Basal Medium and EGM™-2 SingleQuots™ Supplements (Lonza). The cultures were maintained at 37 °C in a humidified atmosphere of 95% air and 5% CO_2_.

During experiments, the cells were plated in culture dishes at high density (80–90% of confluency) and then simultaneously exposed to exogenous UA in various concentrations.

The UA was dissolved in filtered 1M NaOH. The dissolution process was carried out in a thermoblock with shaking (2000 rpm) for 5 min at 70°. There were three UA concentrations: 5 mg/dL, 7.5 mg/dL, and 10 mg/dL. The control group consisted of HUVECs exposed to a culture medium containing the appropriate volume of solvent (NaOH) without the addition of exogenous uric acid (0 mg/dL). Each time the solutions were prepared freshly. To ensure consistent experimental conditions, particularly a stable UA concentration, the culture medium was replaced every 48 h. HUVECs were exposed to UA for 168 h.

The UA concentrations were selected to match those found in the serum of patients with CVDs. The serum UA reference ranges vary by sex. Typically, serum UA levels range from approximately 3 to 15 mg/dL [7]. The exposure time (168 h) was chosen to be as long as possible to ensure that the results reflected the long-term effects of UA on HUVECs. The exposure duration was determined based on the viability of HUVEC cells, as assessed by the MTT assay. The longest time point was selected at which cells exposed to the chosen UA concentrations maintained stable viability levels.

### 2.3. Conditioned Medium (CM) Generation

To generate a CM, young HUVECs were seeded into 25 cm^2^ flasks. When they reached approximately an 80% confluence, they were exposed to exogenous UA for 168 h. Afterwards, the cells were washed with PBS and incubated in serum-free media for 72 h. After this time, the media were collected and the cells that generated the media were counted. All resulting CMs were filtered through a 0.2 μm pore size filter and frozen at −80 °C until use.

### 2.4. Detection of Senescence-Associated β-Galactosidase (SA-β-Gal)

The activity of SA-β-Gal in cell extracts was quantified by measuring the rate of conversion of 4-methylumbelliferyl-β-D-galactopiranose to 4-methylumbelliferone, essentially as described by Gary and Kindell [23]. The activity was quantified with a Synergy™ 2 spectrofluorometer (BioTek Instruments, Winooski, VT, USA).

For cytochemical assessment of SA-β-Gal, the procedure was performed as described in Sosinska et al. [24]. Briefly, cells were cultured on Lab-TekTM Chamber Slides (Nunc, Roskilde, Denmark, cat. no. 177445PK), fixed with 3% formaldehyde (Merck, cat. no. F1635), rinsed and exposed for 2 h at 37 °C to a solution containing 1 mg/mL 5-bromo-4-chloro-3-indolyl-β-D-galactopyranoside (X-Gal, Merck, cat. no. B4252), 5 mM potassium ferrocyanide (Merck, cat. no. P3289), 5 mM potassium ferricyanide (Merck, cat. no. P8131), 150 mM NaCl (Chempur, Piekary Śląskie, Poland, cat. no. 117941206), 2 mM MgCl_2_ (Merck, cat. no. M2393), and 40 mM citric acid (Chempur, cat. no. 115382101) with a pH of 6.0.

### 2.5. Detection of Histone γ-H2A.X and 53BP1

Immunofluorescence of γ-H2A.X and 53BP1 foci was evaluated using methodologies described in [25] with anti-γ-H2A.X (at Ser139) and anti-53BP1 antibodies.

After 48 h and 168 h, cells were fixed with 3% formaldehyde and incubated with a monoclonal antibody against 53BP1 (Novus Biologicals, Abingdon, UK) diluted to 1:500 and γ-H2A.X (Ser 139) (Novus Biologicals, Abingdon, UK), diluted to 1:500 for 1 h at room temperature, respectively. Next, cells were washed and treated with Alexa Fluor 488 goat anti-mouse IgG (Novus Biologicals, Abingdon, UK), diluted to 1:500. After that, cells were stained with 1 μg/mL DAPI. The fluorescence was monitored in a Synergy H1 with excitation at 485 nm and emission at 535 nm. The results were expressed as relative light units (RLU) per 105 cells.

### 2.6. Cell Viability and Proliferation Measurements

Cell viability was assessed using an [3-(4,5-dimethylthiazol-2-yl)-2,5-diphenyltetrazolium bromide] (MTT) assay. HUVECs were seeded in 96-well plates at a high density of 5 × 104 cells/cm^2^ and were allowed to attach overnight. Then, cells were exposed to control and UA-supplemented media for 168 h. Next, the media were removed, cells were washed with phosphate-buffered saline (PBS), and a fresh serum-free medium containing 1.25 mg/mL MTT salt was added for 24 h at 37 °C. After incubation, the generated formazan product was solubilized by adding 20% of sodium dodecyl sulfate and 50% of N, N-dimethylformamide. The absorbance of the converted dye was recorded at 595 nm with a reference wavelength of 690 nm.

Cell proliferation was determined by counting cells in a Bürker chamber. Cells were seeded at a fixed low density and allowed to grow for 168 h. Afterwards, the cells were harvested and counted. The results are expressed as the percentage of cells collected on a given plate relative to the control value (cells not exposed to UA).

### 2.7. Cell Secretome

We selected five cytokines that, according to the literature, may be associated with UA activity and cellular senescence.

Concentrations of CXCL1/GRO-1, CXCL8/IL-8, IL-6, HGF, and TGF-β1 in CM from HUVECs were determined with appropriate DuoSet^®^ Immunoassay Development kits (R&D Systems, Minneapolis, MN, USA) according to the manufacturer’s instructions.

### 2.8. Protocol for Cytokine Detection by ELISA Method

The capture antibody was used at the working concentration in PBS without carrier protein. The plate was sealed and incubated overnight at room temperature. Each well was aspirated, and the plate was washed with a wash buffer. This process was repeated twice for a total of three washes. Each wash was performed by filling the wells with a wash buffer. Complete removal of the liquid at each step was ensured for optimal performance. The plates were blocked by adding 300 μL of reagent diluent to each well and incubated at room temperature for at least 1 h. Aspiration and washing were then repeated. Samples or standards, prepared in reagent diluent or an appropriate diluent, were added to the wells. The plate was covered with an adhesive strip and incubated for 2 h at room temperature. Aspiration and washing were repeated. The detection antibody, diluted in reagent diluent, was added to each well. A new adhesive strip was applied, and the plate was incubated for 2 h at room temperature. Aspiration and washing were again repeated. The working dilution of Streptavidin-HRP was added to each well. The plate was covered and incubated for 20 min at room temperature, avoiding direct light exposure. Aspiration and washing were repeated. The substrate solution was added to each well and incubated for 20 min at room temperature, again avoiding direct light. The stop solution was then added to each well. The absorbance was measured at 450 nm and 540 nm. The difference in absorbance was calculated to correct for optical imperfections in the plate. The results were referenced to the standard curve.

### 2.9. Measurement of Oxidative Stress-Related Parameters

To assess the generation of mitochondrial superoxides and cellular peroxides, MitoSOX red and dihydrorhodamine 123 (DHR) were used. The evaluation of mitochondrial mass was conducted using a staining approach with 10-n-nonyl-acridine orange (NAO). Additionally, the mitochondrial membrane potential (ΔΨm) was determined in cells stained with 1 μM 5,5′,6,6′-tetrachloro-1,1′,3,3′-tetraethylbenzimidazolylcarbocyanine iodide (JC-1).

DHR is an uncharged and nonfluorescent reactive oxygen species (ROS) indicator that can passively diffuse across membranes, where it is oxidized to cationic rhodamine 123, which localizes in the mitochondria and exhibits green fluorescence. A working concentration of DHR123 (30 µm) was used, and cells were incubated at 37 °C for 15 min. Fluorescence was read at 507/540 nm. A working concentration of NAO (10 µm) was used, with incubation at 37 °C for 10 min. Fluorescence was read at 435/535 nm. MitoSOX superoxide indicators are novel fluorogenic dyes specifically targeted to mitochondria in live cells. Oxidation of the MitoSOX red reagent by mitochondrial superoxide produces bright green or red fluorescence. A working concentration of MitoSOX Red (5 µm) was used, and cells were incubated at 37 °C for 10 min. Fluorescence was read at 396/610 nm. The last of the markers used was JC-1. JC-1 is a membrane-permeable cationic dye that is used to study mitochondrial integrity in the context of cellular apoptosis. It selectively enters mitochondria and changes fluorescence characteristics with alteration in mitochondrial transmembrane potential (ΔΨm). In healthy cells with a high mitochondrial ΔΨm, JC-1 forms complexes known as J-aggregates, which fluoresce red/orange. A drop in ΔΨm, a very early event in apoptosis, results in JC-1 monomers, which fluoresce green. A working concentration of JC-1 (10 µm) was used, and cells were incubated at 37 °C for 30 min. Fluorescence was read at 520–570 nm, emission at 570–610 nm for J-aggregates, and excitation at 485 nm with emission at 535 nm for monomers. Detailed protocols for these techniques are provided in the referenced material [19].

### 2.10. Statistical Analysis

Statistical analysis was performed using GraphPad Prism 10.00 software (GraphPad Software, San Diego, CA, USA). One-way ANOVA was employed, followed by the Kruskal–Wallis test for initial assessments and Dunn’s multiple comparison tests for post-hoc analysis. The data are expressed as mean ± SD and a *p*-value < 0.05 was regarded as statistically significant.

## 3. Results

### 3.1. HUVEC Health/Condition, Senescence, and Oxidative Stress-Related Parameters

**Figure 2:**
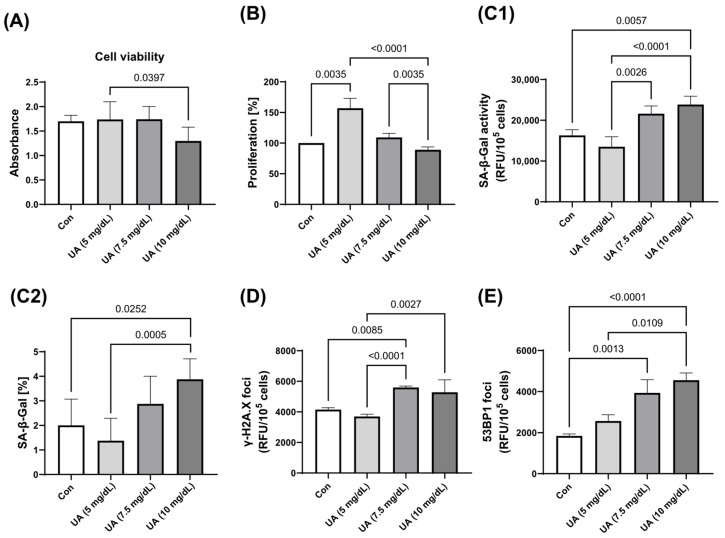
Effect of varying concentrations of exogenous UA on HUVEC (**A**) viability, (**B**) proliferation, and senescence markers. Quantification (**C1**) and qualification (**C2**) of SA-β-Gal activity and the expression of (**D**) γ-H2A.X and (**E**) 53BP1. The data are presented as mean ± SD.

As the concentration of exogenous UA increased, there was a significant decrease in HUVEC viability and an increase in HUVEC proliferation, accompanied by an increase in SA-β-Gal activity and the expression of γ-H2A.X and 53BP1 foci. There was no significant change in HUVEC senescence parameters from 0 to 5 mg/dL UA, but there was a significant acceleration of senescence at UA concentrations of 7.5 mg/dL (Figure 2).

**Figure 3:**
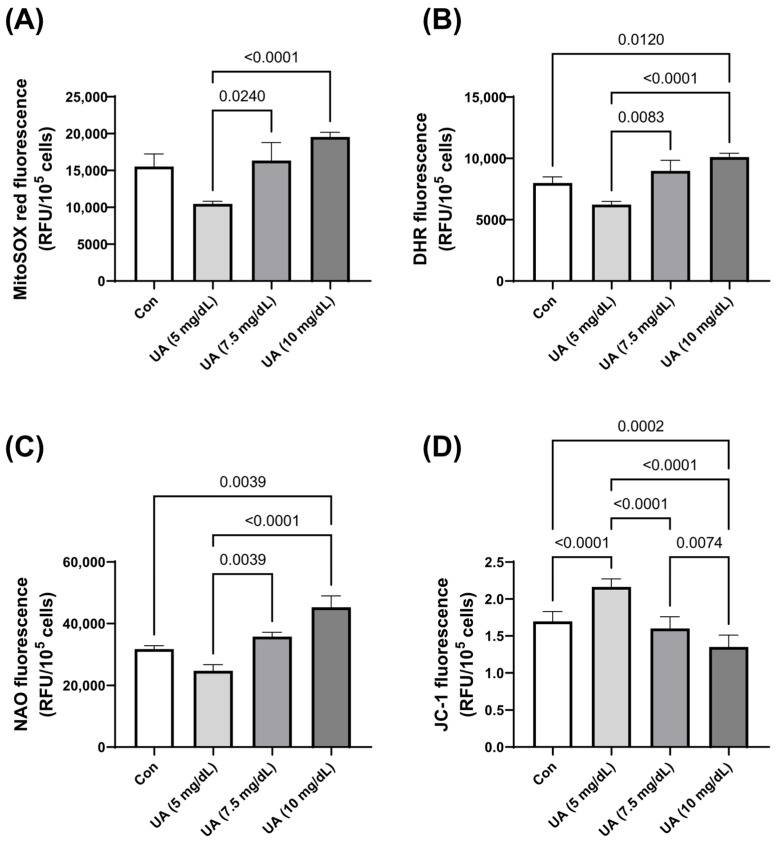
Effect of varying concentrations of exogenous UA on HUVEC oxidative stress-related parameters. Significant alterations were noted in the levels of (**A**) mitochondrial superoxides, (**B**) cellular peroxides, (**C**) mitochondrial mass, and (**D**) mitochondrial membrane potential. The data are presented as mean ± SD.

There was also an increase in the production of mitochondrial superoxides and cellular peroxides, as demonstrated by MitoSOX and DHR fluorescent staining, with increasing UA concentrations. This was accompanied by a higher mitochondrial mass and a decrease in mitochondrial membrane potential (Figure 3). These effects, including mitochondrial dysfunction (increased ROS, NAO and decreased JC-1) and elevated peroxide levels in the cytosol (DHR), may point to potential mechanisms underlying the induction of cellular senescence by UA.

### 3.2. The Assessment of Inflammatory Cytokines and Growth Factors

**Figure 4:**
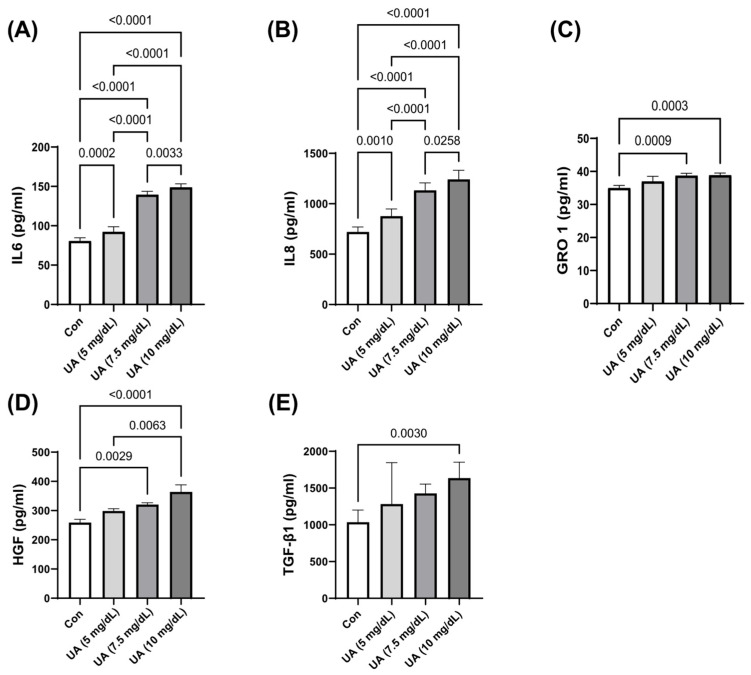
UA-induced secretion of (**A**) IL-6, (**B**) IL-8, (**C**) GRO-1, (**D**) HGF, and (**E**) TGF-β1 by HUVECs. The data are presented as mean ± SD.

HUVEC exposure to UA dose-dependently increased the secretion of IL-6, IL-8, HGF, GRO-1, and TGF-β1 (Figure 4).

## 4. Discussion

Although UA is regarded as an independent risk factor for CVDs, its mechanism of action and impact on endothelial cells remain unclear. Therefore, this study was designed to determine the in vitro effects of UA on HUVEC senescence as a marker of endothelial dysfunction in order to determine the link between UA and CVDs and identify the mechanism(s) involved. The present study demonstrates that in vitro UA, at concentrations of 7.5 mg/dL and above, significantly accelerates senescence in HUVECs, possibly driven by ROS-induced DNA damage. Moreover, UA stimulates the release of cytokines and growth factors involved in inflammation. There was also an increase in cytosolic ROS and mitochondrial damage, as evidenced by a significant increase in cytosolic peroxides and mitochondrial superoxide production, accompanied by a reduction in mitochondrial membrane potential and an increase in mitochondrial mass.

Typically, JC-1 is used to assess mitochondrial membrane potential, a key indicator of the cell’s ability to generate ATP and mitochondrial health [14,26]. The present study reveals that JC-1 decreased with mitochondrial damage [26], and as a compensatory response, the cell produces more healthy mitochondria, reflected by an increase in NAO levels, a marker of mitochondrial mass [27]. NAO levels increased when there was a rise in the number or volume of mitochondria, which could suggest a favorable change. Increased mitochondrial mass does not always indicate improved mitochondrial function, as it can also be a response to mitochondrial damage or dysfunction [28,29,30]. Increased NAO also occurs as a response to various cellular stressors demanding higher energy production. For instance, when factors like free radicals damage mitochondria, cells may increase mitochondrial biogenesis to compensate for the loss of function. Similarly, under oxidative stress, cells boost mitochondrial mass as an adaptive response, often accompanied by increased ROS production. Hypoxic conditions also trigger increased mitochondrial numbers to better utilize the available oxygen. Additionally, more mitochondria may form in senescent cells to compensate for declining mitochondrial function and maintain essential cellular processes [28,29,30,31].

The present study demonstrates that high concentrations of exogenous UA induce HUVEC senescence with exogenous UA at concentrations of 7.5 mg/dL and above, significantly reducing HUVEC viability and increasing proliferation. This was accompanied by a marked elevation in SA-β-Gal activity and γ-H2A.X and 53BP1 expression, indicating accelerated cellular senescence. The results, therefore, indicate adverse changes characteristic of cellular senescence. The in vitro studies published so far are largely consistent with our results.

In HUVEC incubated with a UA concentration of 6 mg/dL and higher for 48 h, a significant increase in the level of SA-β-Gal was observed, serving as a marker for the senescence process. At concentrations equal to or above 9 mg/dL, the authors observed decreased proliferation and increased apoptosis [20]. Cellular senescence assessment was only based on one senescence marker (SA-b-Gal), which is not universally applicable. It should not be interpreted in isolation without considering other markers of senescence [32]. Li et al. showed that HUVEC incubated with UA at a concentration of 5 mg/dL for 24 h resulted in an antioxidant effect, reversing H_2_O_2_-induced senescence, while a concentration of 10 mg/dL was harmful to cells and induced senescence (an increased level of senescence-related markers: SA-β-Gal, p16 and p21, and TNF-α, IL-6, ROS) [21]. Wu et al. demonstrated that elevated UA levels lead to inhibition of HUVEC proliferation, viability, and induction of cellular senescence (an increase in senescence markers) and apoptosis. They investigated selected markers of senescence, such as SA-β-Gal, levels of p53, p21, and p16 [22]. Despite the proper identification of senescent cells, this study has several limitations. First, the focus was primarily on high UA concentrations (50 mg/dL), which may not reflect the commonly encountered physiological levels in the human body, limiting the broader clinical applicability of the findings. Furthermore, the limited range of UA concentrations prevents the identification of the threshold at which UA begins to exert pro-senescence effects. Secondly, the study examined relatively short exposure times to UA (up to 72 h), which may not capture the full, long-term effects of UA on endothelial cells. In contrast, the present study investigated the longer effects of clinically relevant UA concentrations. Based on the above, UA induced cellular senescence in HUVECs, and the possible mechanism is based on the so-called free radical theory, whereby ROS induces cell damage. This theory suggests that ROS-induced DNA damage is the main and initial driver of cellular senescence [33].

The increase in inflammatory cytokines and growth factors observed in the present study indicate a shift in the secretory profile of HUVECs towards that of senescent cells. The characteristic of senescent cells is the presence of the secretory phenotype, also known as the SASP or senescence-messaging secretome (SM). SASP includes components such as cytokines, proteases, growth factors, and substances that act through autocrine or paracrine signaling [17]. The observed increase in the secretion of all the examined cytokines and growth factors by HUVECs with increasing UA concentrations demonstrate the negative impact of UA on cells. The excessive production of pro-inflammatory factors adversely affects the vascular endothelium, exacerbating dysfunction. However, this increase could represent an entirely distinct cellular response to the stimulus posed by UA. Inflammation induced by UA likely occurs through the activation of inflammatory markers such as IL-6, IL-8, GRO-1, and, to a lesser extent, TGF-β1. Cai et al. demonstrated that in vitro UA at a concentration of 20 mg/dL induces inflammatory cytokines involved in the pathogenesis of HUVEC dysfunction by showing a significant increase in IL-6 and TGF-α levels (exposure time: 0, 12, 24, 48 h) [34]. Also, in vitro UA at a concentration of 12 mg/dL caused a significant increase in IL-8 in HUVECs after 6 h of exposure [35]. The secretion of key SASP cytokines, such as IL-6, is significantly increased in response to DNA damage [36]. Although IL-8 is often interpreted as a ‘bad agent’, it may have protective functions, as Shen et al. demonstrated that IL-8 exerted protective effects against endothelial senescence, potentially due to telomerase activation [37].

GRO-1 (CXCL1) is a key player in regulating inflammation and immune responses in senescent cells. As part of the SASP, it promotes abnormal chronic inflammation by attracting neutrophils and other immune cells, a hallmark of aging. This signaling reinforces the senescent state and can spread to neighboring cells, contributing to tissue dysfunction and age-related diseases [38]. In endothelial cells like HUVECs, GRO-1 exacerbates vascular dysfunction by promoting inflammation and impairing their regenerative capacity. Wang et al. suggested that understanding the pathways associated with GRO-1 could serve as a potential mechanism for future treatment of patients with elevated UA levels [39].

We observed an increase in TGF-β1. Although it is a typical marker characterizing the senescent cell phenotype [40], its role appears to be smaller compared to the other parameters studied. It is elevated in the serum of patients with hyperuricemia and induces inflammation [41]. It may play a larger role in the presence of additional factors, while in our model, UA alone is insufficient.

In senescent cells, HGF plays a more complex role, supporting tissue regeneration by promoting cell repair, but its function is limited in aging tissues. HGF is also a marker of vascular stiffness, reflecting vascular dysfunction [42,43]. Nakamura et al. showed that HGF could protect and repair vascular endothelial cells damaged by high blood pressure and observed a significant increase in serum HGF levels correlated with the severity of hypertension [43]. In light of our research, the increase in HGF linked to UA levels in the serum might be regarded as a protective response of endothelial cells. Despite the increased HGF release by HUVECs, they cannot stop the progressive damage.

All the markers mentioned above are important inflammatory markers, but unfortunately, their limited number does not allow us to determine whether they are key components of signaling pathways or if their elevated levels represent a UA-induced SASP.

In summary, it is widely acknowledged that in vitro experiments should closely mimic conditions that could occur in vivo. The harmful impact of UA, which triggers cellular senescence and causes vascular endothelial dysfunction, plays a role in the onset of atherosclerosis and, as a result, contributes to the development of CVDs [44].

Limitations

This study has certain limitations. First, exogenous UA was used, which at high concentrations may form UA crystals, potentially leading to lower UA levels in the culture medium. To prevent this, all UA solutions were freshly prepared, and each batch was checked for precipitation. Another important aspect concerns the use of NaOH to dissolve UA. Although UA is naturally water-soluble, NaOH was the only solvent to achieve complete dissolution. It is worth noting that NaOH has also been used by other researchers for this purpose [17]. We also confirmed that NaOH added to cell cultures at the concentration used to dissolve UA did not affect cell viability. Furthermore, only a limited set of parameters were assessed, and a broader panel might have provided a more comprehensive insight into the mechanisms by which UA influences cellular senescence, which could have led to additional conclusions.

## 5. Conclusions

Increasing UA concentrations (7.5 mg/dL and higher) significantly promotes HUVEC senescence, possibly due to ROS-induced DNA damage. Furthermore, UA stimulates the production of cytokines and growth factors associated with inflammation.

## Data Availability

The original work presented in the study is featured in the article, and any additional questions can be directed to the corresponding author.

## Abbreviations

The following abbreviations are used in this manuscript:

CVD	Cardiovascular disease
SASP	Senescence-Associated Secretory Phenotype
SM	Senescence-Mediated
Il-6	Interleukin 6
Il-8	Interleukin 8
GRO-1	Growth-Regulated Oncogene 1
TGF- β1	Transforming Growth Factor Beta 1
HGF	Hepatocyte Growth Factor
JC-1	5,5′,6,6′-Tetrachloro-1,1′,3,3′-tetraethylbenzimidazolylcarbocyanine Iodide
NAO	Naphthalene 2,3-dicarboxaldehyde
CVD	Cardiovascular Disease
ROS	Reactive Oxygen Species
XO	Xanthine Oxidase
ESH	European Society of Hypertension
UA	Uric Acid
HUVEC	Human Umbilical Vein Endothelial Cells
SA-β-Gal	β-Galactosidase
CM	Conditioned medium
SM	Senescence-messaging secretome
